# Parkinson's disease and dopamine transporter neuroimaging – a critical review

**DOI:** 10.1590/S1516-31802006000300014

**Published:** 2006-05-04

**Authors:** Ming Chi Shih, Marcelo Queiroz Hoexter, Luiz Augusto Franco de Andrade, Rodrigo Affonseca Bressan

**Keywords:** Parkinson disease, Dopamine, Emission-computed tomography, Single-photon emission-computerized tomography, Diagnosis, Doença de Parkinson, Dopamina, Tomografia computadorizada de emissão, Tomografia computadorizada de emissão de fóton único, Diagnóstico

## Abstract

Parkinson's disease (PD) is a common neuro-degenerative disorder that is mainly caused by dopaminergic neuron loss in the substantia nigra. Several nuclear medicine radiotracers have been developed to evaluate PD diagnoses and disease evolution in vivo in PD patients. Positron emission tomography (PET) and single photon computerized emission tomography (SPECT) radiotracers for the dopamine transporter (DAT) provide good markers for the integrity of the presynaptic dopaminergic system affected in PD. Over the last decade, radiotracers suitable for imaging the DAT have been the subject of most efforts. In this review, we provide a critical discussion on the utility of DAT imaging for Parkinson's disease diagnosis (sensitivity and specificity).

## RESUMO

A doença de Parkinson (DP) é uma desordem neurodegenerativa causada por perda de neurônios dopaminérgicos na substância negra. Vários traçadores da medicina nuclear têm sido desenvolvidos para avaliar o diagnóstico e acompanhamento da DP. Traçadores para o transportador de dopamina (TDA) utilizados na tomografia por emissão de pósitrons (PET) e tomografia por emissão de fóton único (SPECT) demonstram boa marcação na integridade de sistema dopaminergico pré-sináptico, afetada na DP. Na última década, radiotraçadores apropriados para imagens de TDA têm sido mais estudados. Nesta revisão, provemos uma discussão crítica sobre a utilidade dessas imagens de TDA para o diagnóstico de DP (sensibilidade e especificidade).

## INTRODUCTION

Parkinson's disease (PD) is a common neurodegenerative disorder characterized by the presence of Lewy bodies and progressive degeneration of dopaminergic neurons in the *substantia nigra*, with loss of their nerve terminals in the basal ganglia structures, especially in the striatum.^1^ Other etiopathogenic processes are suspected to incite and perpetuate PD, such as oxidative stress, mitochondrial dysfunction, proteasome dysfunction and protein aggregation, which can lead to nigrostriatal cell dysfunction and death.^2^

The overall prevalence of PD is estimated at 0.2% but rises with increasing age, affecting as many as 0.5-1% of individuals aged 65-69 years and as many as 1-3% of individuals older than 80 years.^3^ PD diagnosis is substantially based on clinical symptoms and is characterized by resting tremor, rigidity, bradykinesia, and postural instability, and also favorable response to levodopa therapy,^1,4^ but few researchers have attempted to develop rigorous diagnostic criteria that can be applied consistently and assessed for reliability.^1^

To address the problem of identifying Parkinson's disease in its initial phases of clinical expression, three levels of diagnostic confidence have been differentiated: definite, probable and possible.^1,5^ The diagnoses of possible and probable PD are based on clinical criteria alone, whereas the presence of Lewy bodies in histopathological findings is required for definite PD diagnosis. However, Lewy bodies are also present in many other diseases^6,7^ and are probably absent in autosomal recessive juvenile Parkinsonism,^8^ which makes these diagnostic criteria not totally satisfactory.^9^

In clinical-pathological studies, up to 25% of cases with an antemortem clinical diagnosis of PD were found not to have PD at postmortem examination.^1,10^ Parkinson's disease patients manifest symptoms only when 50 to 80% of the nigrostriatal neurons are lost. Clinical diagnosis fails to identify individuals before they reach such a significant loss of dopamine neurons. Such individuals would benefit from early diagnosis, before dopamine loss is too severe, with the aim of attempting to implement the neuroprotective interventions that have been developed recently. Thus, improvement in the accuracy of clinical diagnoses of PD is needed for epidemiological studies and practical clinical treatment.^1,11^

In this review, we discuss the relationship between the dopamine system, especially the dopamine transporter (DAT), and Parkinson's disease (PD). We also discuss the usefulness of DAT neuroimaging using positron emission tomography (PET) and single photon emission computerized tomography (SPECT) for PD diagnosis and follow-up.

### Molecular imaging Molecular imaging

Conventional high-resolution imaging techniques such as magnetic resonance imaging (MRI) and computed tomography (CT) are not so useful for PD diagnosis. These techniques do, however, have a role in the differential diagnosis with some other types of Parkinsonism. Researchers have been interested in developing sensitive diagnostic techniques for early PD by assessing DAT concentrations in the striatum. The key to molecular imaging in nuclear medicine is radiotracers: substances that have high affinity and specificity to a receptor site that is labeled with a radioisotope. These radioligands allow *in vivo* evaluation of receptor density and affinity, measured as binding potential.^12^ The tracers are labeled with [^123^I] and [^99m^Tc] for SPECT, or isotopes [^11^C], [^18^F], [^15^O] and [13N] for PET. Although PET provides higher resolution and better physical quantitative capacity than SPECT, PET is less practical as a routine procedure because of its high cost and the shorter half-life of its radiotracers. On the other hand, SPECT uses isotopes with longer half-lives that can be stored on site.

Over the last decade, radiotracers suitable for DAT imaging have been the subject of great investigative efforts. Several DAT ligands have been successfully used as methods for evaluating neuronal loss, for PD diagnosis.^11,13–19^ For this reason, DAT ligands have become well-established markers that are useful for evaluating changes in presynaptic DAT sites *in vivo* and *in vitro*.

### Dopamine transporter (DAT)

Dopamine transporter (DAT) was first described 30 years ago. It is an 80-kDa protein composed of 12 transmembrane spanning regions with the carboxyl and amino termini residing intracellularly.^20^ The protein is externally glycosylated and is localized in the axonal membranes of nigrostriatal dopaminergic neurons.^21^ The human DAT gene is localized on chromosome 5p15.3.^22–24^ DAT is located on the plasma membrane of nerve terminals in a small number of neurons in the brain, especially in the striatum and nucleus accumbens, but also in the globus pallidus, cingulate cortex, olfactory tubercle, amygdala and midbrain.^25^ DAT regulates the dopamine concentration in the synaptic cleft through reuptake of dopamine into presynaptic neurons; it plays a central role in the spatial and temporal buffering of the released dopamine.^26^ Its activity can be regulated by presynaptic receptors, protein kinases, and membrane trafficking.^27–29^ DAT exerts vital influence on dopamine function, by modulating locomotor activity, cognition and the reward system.^30^ Pharmacologically, DAT serves as the binding site for drugs of abuse^31^ (e.g. cocaine and amphetamine) and therapeutic agents^32^ (e.g. methylphenidate and bupropion). It has been observed that striatal DAT declines at a rate of approximately 6-7% per decade in the human striatum.^33–35^ The density of DAT can be used as a marker for dopamine terminal innervation.^36^

### DAT neuroimaging

Postmortem studies have shown an association between PD and striatal DAT concentration.^37,38^ Evaluation of DAT in human postmortem tissue has demonstrated good correlation of *in vitro* tracers such as [^125^I] altropane and DAT reduction in PD.^39^ DAT provides a good site for monitoring the integrity of the presynaptic dopaminergic systems that are most affected in PD. Several DAT agents have been developed for diagnosing PD and monitoring the treatment of PD patients, based on DAT antagonists such as methylphenidate and cocaine (tropane derivatives).^18,40,41^ Cocaine analogs (WIN35428) and their derivatives gain potency through halogenation of their phenyl rings. On the other hand, lack of ester linkages between tropane and phenyl moiety sites is the main mechanism for cocaine inactivation and degradation. Table 1 summarizes the DAT radiotracers that have reached phase III or IV of clinical applications, including [^11^C] cocaine, [^123^I] β-CIT (2b-carboxymethoxy-3b-[4-iodophenyl] tropane), [^123^I] FE-CIT (ioflupane), [^123^I]/[^18^F]/[^11^C] FP-CIT (N-[3-fluoropropyl]-2ss-carbomethoxy-3ss[4-iodophenyl]nortropane), [^18^F]/[^11^C] CFT (2beta-carbomethoxy-3betafluorophenyl-tro-pane), [^123^I]/[^11^C] altropane, [^123^I]/[^11^C] PE2I (N-{3-iodoprop-(2E)-enyl}-2beta-carboxymethoxy-3beta-{4'methylphenyl} nortropane), and [^11^C] methylphenidate.

**Table 1.** Dopamine transporter radiotracers according to the literature

Tracer	Isotope	Scan time (min.)	Peak striatal activity (min.)	Signal to noise ratio	SERT: DAT	PET/SPECT	References
Cocaine	[^11^C]	10-20	4-7	< 2	Almost equal affinity	PET	Ritz et al., 1990^45^ Logan et al., 1990^46^ Telang et al., 1999^47^
CFT	[^11^C]	60-90	225	5	>12-fold Ki 12.6	PET	Frost et al., 1993^13^ Rinne et al., 1999^48,49^ Madras et al., l989^50^ Morris et al., 1996^51^ Haaparanta et al., 1996^52^ Brownell et al., 1996^53^
CFT	[^l8^F]	120	225	10	4-l6 DAT:SERT	PET	Haaparanta et al., 1996^52^
WIN 35,428	[^3^H]	NA	NA	NA	NA	PET	Gatley et al., 1995^54^
β-CIT or RTI 55 dopa scan	[^123^I]	1440	900-1200	9	1.6	SPECT	Innis et al., 1993^19^ Laruelle et al., 1994^55^ Brucke et al., 1993^56^ Laruelle et al, 1994^57^
FE or FP-CIT	[^123^I] [^11^C] [^18^F]	70-240	30	8	Same β-CIT	PET: SPECT	Kuikka et al., 1995^58^ Antonini et al., 2001^59^ Tissing et al, 1998^60^ Booij et al., 2001^61^ Lundkvist et al., 1997^62^ Chaly et al., 1996^63^ Abi-Dargham et al., 1996^64^
Altropane	[^123^I] [^11^C]	60-1 20	10-15	~6	25-fold DAT. Sert selectivity Ki 6.8	SPECT PET	Madras et al, 1998^39,65,66^ Fischman et al., 1998^67^ Elmaleh et al., 1996^68^
PE2I or RTI-32	[^123^I]	8 -70	~70	8	high affinity (Ki =17 nM) and selectivity for DAT	SPECT PET	Emond et al., 1997^69^ Chalon et al., 1999^70^ Hall et al., 1999^71^ Guilloteau et al., 1998^72^ Poyot et al., 2001^73^ Kuikka et al., 1999^74^ Repo et al., 1999^75^
TRODAT-1	[^99m^Tc]	240	180-240	2.5	Ki = l2 nM	SPECT	Choi et al., 1999^76^ Acton et al., 1999^77^ Kao et al., 2001^78^
D-threo-methylphenidate	[^11^C]	30	15	2-3	Ki = 12 nM DAT	PET	Gatley et al., 1995^54^ Volkow et al., 1995^32^ Lee et al., 2000^79^

*CFT = 2beta-carbomethoxy-3betafluorophenyl-tropane;WIN = [11C]2beta-carbomethoxy-3beta-(4-fluorophenyl)-tropane;* β*-CIT = 2b-carboxymethoxy3b-[4-iodophenyl] tropane; RTI = [1R-(exo,exo)]- 3-[4-(iodo123)phenyl]-8-methyl-8-azabicyclo[3.2.1]o ctane-2-carb oxylic acid methyl ester; FE-CIT = ioflupane; FP-CIT = N-[3-fluoropropyl]-2ss-carbomethoxy-3ss-[4-iodophenyl]nortropane; PE2I = N-{3-iodoprop-(2E)-enyl}-2beta-carboxymethoxy-3beta-{4'methylphenyl}nortropane; RTI-32 = methyl (1R-2-exo-3-exo)-8-methyl-3-(4-methylphenyl)-8-azabicyclo[3.2.1]octane-2-carboxylate; TRODAT-1 = [2-[[2-[[[3-(4-chloro- phenyl)-8-methyl-8-azabicyclo[3, 2, 1]oct-2-yl]methyl] (2-mercaptoethyl)amino]ethyl]amino]ethanethiolato(3)]-oxo-[1R-(exo -exo)]; NA = not available; DAT = dopamine transporter; SERT = serotonin transporter; PET = photon emission tomography; SPECT = single photon emission computerized tomography.*

Previous molecular imaging approaches with [^18^F] dopa and PET labeling for dopadecarboxylase (the enzyme involved in dopa-mine synthesis) was considered to be the gold standard for evaluating nigral dopaminergic neurons in PD^42,43^ before the advent of DAT tracers. Tropane derivative studies ([11C] CFT and [^123^I] β-CIT) have shown a direct correlation between decreased DAT in the putamen and PD symptoms.^13,44^ Descriptions of the characteristics of several DAT tracers, including time to reach tracer equilibrium (scan time); tracer maximum accumulation (peak striatal activity); differential contrast imaging in the striatal area and the rest of the brain, especially in the cerebellum (signal to noise ratio); and affinity competition inhibition of serotonin and dopamine transporters (SERT: DAT) are given in Table 1.^13,19,32,39,45–79^

#### DAT quantification technique

Region-of-interest (ROI) semi-quantitative evaluation techniques have to be used to assess specific DAT binding in the striatum and over its subregions (head of caudate and putamen). The investigator performing the ROI analyses needs to be unaware of the subject's demographic characteristics.^80^ Transverse/oblique slices are usually chosen for ROI definition, such as transaxial slices oriented along the orbitomeatal line, and the two slices corresponding to the highest right and left striatal uptake are positioned on summed images.^81^ Data evaluation must always consider relevant morphological information (by CT or MRI), especially in structural lesions in the basal ganglia and the reference structures that are chosen as ROI. Given that postmortem studies have shown a very low density of DAT and SERT in occipital cortices and the cerebellum,^82–84^ these reference regions with absent (or low) DAT density are taken to indicate nondisplaceable DAT activity and are used to assess nonspecific binding. Quantitative studies that are coregistered to the template or performed with anatomically adjusted ROIs (using templates or MRI overlay techniques) or on a pixelwise basis, in which they self-correspond exactly to the three-dimensional ROI map, ensure that the results are highly observer-independent, precise, and reproducible, because of the automated processing.^85^ Striatal dopamine ROI areas for monoamine transporters, mainly reflecting SERT,^82^ are visually positioned on the summed transversal slices of the hypothalamus/midbrain (including the raphe nuclei, substantia nigra and colliculi), the thalamus and the medial prefrontal area at the striatal level. These areas have been identified through magnetic resonance imaging scans using a reference atlas.^86^ If available, ROI definition may be based on individual morphology, as obtained by image fusion with MRI, which is particularly important when low specific binding is expected (e.g. in cases of severe loss or blockade of the DAT).

DAT binding potential (BP), corresponding to the product of the free receptor density and affinity, is calculated as the ratio of striatal specific binding to steady-state free unmetabolized plasma tracer concentration.^87^ Given that each tracer attains a state of equilibrium in the striatal and occipital areas at a certain time after its injection, the ratio point at this time is used as an estimate for the BP.^88^ The specific DAT BP in the basal ganglia is calculated as the difference between striatal activity and the reference region, i.e. the occipital activity (OA) at equilibrium. The ratio of the total binding in the striatum minus the nondisplaceable binding in the OA divided by the OA reflects the specificto-nondisplaceable binding.

Before proceeding with imaging quantification, it is necessary to be aware of possible technical artifacts (pitfalls) such as head motion, attenuation artifacts and technical artifacts due to gamma camera problems.

Before interpreting the data, possible interaction with concomitant medications must be taken into account and it is essential to objectively assess the semi-quantification of striatal DAT binding. Inter-individual quantitative results are based on comparisons between specific DAT BP obtained in the patients and normal controls that are preferably age-matched (thereby avoiding over-interpretation: age-dependency is a known pitfall/source of error). Age-specific sensitivity and specificity of DAT SPECT imaging for differentiating patients with PD from healthy subjects is greater than when there is no age matching.^89^

It is also important to utilize the same type of camera and the same image evaluation technique. If age-matched data comparisons are available, it is recommendable to use analytical approaches based on stereotactic normalization, in order to determine abnormalities of DAT BP in an observer-independent manner. Moreover, it is important to establish a control group for the central database, thus allowing comparative calculations for the different imaging protocols.

### Limitations of DAT imaging Limitations of DAT imaging

In routine clinical practice, even experienced neurologists have difficulty in differentiating early-stage PD from atypical Parkinsonian syndromes (APS).^90,91^ The accuracy of the clinical diagnoses of PD, multiple system atrophy (MSA), progressive supranuclear palsy (PSP), cortical basal degeneration (CBD) and vascular Parkinsonism is imperfect and error rates can be as high as 25%.^4^ Although DAT imaging is the best parameter for evaluating dopamine neuron loss and differentiating between Parkinsonism and non-Parkinsonism, it may not be useful for differentiating PD from APS, PSP, MSA, CBD and drug-induced Parkinsonism.^92,93^ Postsynaptic dopamine receptor imaging using PET-SPECT or MR-based techniques such as diffusion weighted imaging or volumetry are more likely to contribute to a differential diagnosis between PD/APS and MSA/PSP than is DAT-SPECT imaging.^94,95^ Although there is controversy in the literature, merged imaging between DAT and MRI may be helpful for differentiating between PSP and PD in routine clinical practice.^96^ Combining this with D2 receptor imaging can differentiate between MSA and PD,^97^ because each type of Parkinsonian syndrome has its own physio-pathology and rates of dopamine neuron loss and post-synaptic receptor compensation in the striatum (caudate and putamen). In contrast, the distinction between CBD and other Parkinsonian syndromes by means of DAT and D2 receptor imaging is limited.^95^

### DAT neuroimaging and PD diagnosis

The most-used radiotracer is [^123^I] β-CIT. In an evaluation of 113 PD patients, there was a good correlation between DAT density loss and PD symptoms (Unified Parkinson's Disease Rating Scale, UPDRS), and the decrease in signal ranged from 35% in a Hoehn-Yahr (H&Y) stage I patient to 75% in an H&Y stage V patient.^92^ Using [^123^I] β-CIT, a multicenter study found sensitivity of 98% and specificity of 83% for PD diagnosis, in differentiation from PSP and essential tremor (ET).^39^ A six-month follow-up study on 35 suspected PD patients found that [^123^I] β-CIT was more accurate (sensitivity 0.92; specificity 1.00) than the clinical diagnosis (sensitivity 0.92; specificity 0.30).^98^ A retrospective study on 72 early-stage untreated Parkinsonian syndrome (PS) patients revealed lower caudate nucleus binding ratios and higher putamen binding ratios among cases that were later diagnosed or rediagnosed as APS and IPD, thus showing that striatal involvement appeared to have little predictive value in these individual cases.^99^

In a study on the fast pharmacokinetic radioligand FP-CIT, 95% sensitivity and 93% specificity for differentiating between Parkinsonism and ET were found.^100^ This provides further evidence that FP-CIT provides robust estimates of disease severity, correlating with the duration of PD.^101^ However, variability in uptake values suggests that factors other than nigrostriatal degeneration may contribute towards disease severity. There is a correlation with bradykinesia but not with tremor, thus suggesting that the origin of tremors is beyond the DAT system. Another study comparing 18 PD patients with or without the Parkinsonism gene has now found that Parkinsonism-related disease may be associated with a higher degree of nigrostriatal impairment, independent of the clinical severity of the disease, and more symmetrical involvement than in non-Parkinsonian early-onset disease.^102^

Imaging studies using the technetium-labeled radioligand TRODAT-1 have found good correlation between the H&Y and UPDRS scales.^103^ This method is useful for early PD detection,^104^ and it has been suggested that it may be useful for differential diagnosis of some kinds of movement disorders.^105^ Results from a crossover study have suggested that [^99m^Tc]-TRODAT-1 SPECT may provide a reliable alternative to [^18^F]-dopa PET in the evaluation of clinical PD patients.^106^ A reproducibility study using TRODAT-1 scans from 20 PD patients showed excellent test-retest reliability in evaluating PD progression.^107^ The diagnostic accuracy of [^99m^Tc]-TRODAT-1 SPECT showed sensitivity of 0.79 and specificity of 0.92 in distinguishing 29 patients with early PD from 38 healthy volunteers.^108^ Furthermore, in a sample of patients with different stages of PD and healthy controls, another research group found good concordance when visual interpretation of [^99m^Tc]-TRODAT-1 SPET images was used to evaluate the presence of PD (sensitivity 0.98 and specificity 0.86).^109^ Using age-matched PD patients and healthy controls, Weng et al. (2004) showed that [^99m^Tc]-TRODAT-1 SPECT has a high sensitivity and specificity for measuring the decrement of DAT in PD patients.^110^ A cross-sectional study on 96 early-stage patients comparing [^123^I] FP-CIT and [^99m^Tc] TRODAT-1, found sensitivity/specificity of 0.95/0.86 and 0.92/0.70, respectively.^111^

There are only a few studies in the literature involving other tropane derivatives, and so far only preliminary experiences have been presented. [^18^F] β-CFT was found to be a sensitive marker for dopaminergic dysfunction that could be used in diagnoses, disease severity assessments and patient follow-up.^47^ Reduced striatal dopamine transporter binding assessed by [^123^I]IPT in patients with early Parkinson's disease demonstrates the potential of this method to detect preclinical disease.^112^ Prunier et al. (2003), studying nonhuman primates chronically treated with 1-methyl-4-phenyl-1,2,3,6-tetrahydropyridine according to a regimen that consistently produces a progressive Parkinsonian state, have shown that [^123^I]-PE2I SPECT was able to detect presymptomatic lesions of nigrostriatal neurons and suggested that this method could now be used clinically for early diagnosis of Parkinson's disease.^113^

## DISCUSSION

Several molecular imaging techniques have been developed for diagnosing and evaluating PD progression. DAT radiotracers seem to be the best markers for identifying PD patients, with high sensitivity and specificity. DAT reduction correlates with dopamine neurons loss in the substantia nigra and striatum.^26,114,115^ This is why the striatal concentration of DAT, preferentially putamen DAT, is a highly sensitive parameter for detecting early phases of PD.^39^ DAT ligands are well-established markers that are useful in evaluating changes in presynaptic DAT sites *in vivo* and *in vitro*.

The PET isotopes and [^123^I] are produced in cyclotrons and have limited availability and relatively high cost, thus limiting the availability of such DAT tracers for routine application in DAT imaging^106^ Studies have reported that [^99m^Tc]-TRODAT-1 SPECT is useful.^105,116^ The ready availability and ease of use of [^99m^Tc] agents using a modified TRODAT-1 preparation kit^117^ are advantages that provide a powerful incentive for their routine use in clinical studies.

Although PD is characterized by selective loss of dopamine neurons in the basal ganglia and substantia nigra, these are not the only brain changes occurring in these patients’ brains. Several other changes leading to neuro-psychological deficits cannot be explained by dopamine loss.^118^ In a study on 32 PD patients using [^123^I] β-CIT, it was found that although striatal uptake was correlated with clinical severity, the annual percentage loss of striatal uptake did not correlate with the annual loss in measurements of clinical function.^119^ On the other hand, there was a non-significant difference in progression rate across three scans obtained over a five-year period among 24 early PD patients.^120^ In a longitudinal study assessing PD progression, the annual rate of reduction of striatal DAT uptake was approximately 6 to 13% in PD patients, compared with 0 to 2.5% in healthy controls, which was in line with the results from [^18^F] dopa-PET studies.^121–123^ However, most of these studies were conducted among patients with advanced PD.^124^ Furthermore, DAT tracers present certain problems in evaluating PD progression, because of changes in the DAT system (up or downregulation) induced by drug treatment, and this method has not been fully validated as an outcome measurement for trials on PD treatments. Future studies should focus on the early stages of PD, i.e. the time when the diagnosis is uncertain and DAT imaging techniques would be more useful.

As indicated above, DAT imaging can only show the dopamine neuron degeneration process, which indeed is the main physiopathology of PD as far as the early stages of the disease are concerned. In fact, few studies have evaluated the usefulness of DAT imaging for providing clear diagnosis in the early stages of PD. Further studies examining the specificity of the diagnosis of PD using DAT imaging are needed. Nevertheless, early dopamine neuron loss can easily be detected by means of SPECT and PET DAT quantification. Early dopamine neuron loss detection enables interventions for minimizing and stabilizing these progressions by means of neuroprotection treatments. Although neuroprotective therapy is still controversial and so far non-existent for PD, several strategies have been studied and it is hoped that they will soon be in use. These strategies include exposure to enriched environments (a combination of exercise, social interaction and learning);^125^ the use of MAO-inhibitors;^126^ L-dopa coadministered with the adenosine A2A receptor agonist;^127^ and gene neuroprotective therapy models (neurotrophic factors, i.e. genes to prevent apoptosis or detoxify free radical species that protect and restore the nigrostriatal pathway).^128^

### Molecular imaging in Brazil

PET isotopes and SPECT ^123^I are produced in cyclotrons and have limited availability, relatively short half-life and high cost, thereby limiting the accessibility of DAT tracers.^106^ In Brazil, the use of those cyclotron-generated radioligands is limited by the volume of tracer production, which is restricted to certain government institutions.^129^ This restriction limits the availability of radiotracers and, more importantly, inhibits the development of the radiopharmacy laboratories and radiochemists that are fundamental for the advance of molecular imaging in Brazil.^130^

The advantage of tracers such as [^99m^Tc] TRODAT-1 is the ready availability and ease manipulation of ^99m^Tc using a modified preparation of TRODAT-1 by means of a kit.^117^ In Brazil, some animal studies using [^99m^Tc] TRODAT-1 have been conducted.^131^ Our group performed the first studies on humans using this tracer and obtained encouraging results. The data so far suggests that this tracer is an uncomplicated and reliable tool for routine clinical studies and favors its general introduction. As noted, the application of these methods in our country is very recent. Today, the first neuroimaging pilot study on humans, using TRODAT-1 and SPECT, is underway in Brazil for evaluating PD patients.^132^ Several other research protocols using [^99m^Tc] TRODAT-1 and SPECT may be developed to investigate DAT in neuropsychiatric disorders involving the dopaminergic system, such as early onset Parkinson's disease, attention deficit and hyperactivity disorder (ADHD), obsessive-compulsive disorder (OCD), post-traumatic stress disorder (PTSD) and schizophrenia.

## CONCLUSIONS

DAT imaging can be used in the differential diagnosis between Parkinsonian and non-Parkinsonian syndromes and in cases of uncertainty involving essential tremor. The high sensitivity and specificity of SPECT semi-quantitative images makes this method a useful tool in the early clinical PD evaluation and in preclinical screening for asymptomatic patients. At the present moment, DAT imaging is the best biomarker for evaluating dopamine neuron loss, which is responsible for most of the motor symptoms in PD patients.

TRODAT-1 is a SPECT tracer that is easy to manipulate. Recent data suggest that it can be safely used in Brazil.^131–133^ Although the current availability is restricted to research centers, it is expected that it will soon be available for routine clinical use.
